# Analysis of the Proteinaceous Components of the Organic Matrix of Calcitic Sclerites from the Soft Coral *Sinularia sp.*

**DOI:** 10.1371/journal.pone.0058781

**Published:** 2013-03-14

**Authors:** M. Azizur Rahman, Ryuichi Shinjo, Tamotsu Oomori, Gert Wörheide

**Affiliations:** 1 Department of Earth and Environmental Sciences, Palaeontology and Geobiology, Ludwig-Maximilians-Universität München, München, Germany; 2 Department of Physics and Earth Sciences, University of the Ryukyus, Okinawa, Japan; 3 Department of Chemistry, University of the Ryukyus, Okinawa, Japan; 4 Bavarian State Collections of Palaeontology and Geology, Munich, Germany; 5 GeoBio-Center, Ludwig-Maximilians-Universität München, München, Germany; University of New South Wales, Australia

## Abstract

An organic matrix consisting of a protein-polysaccharide complex is generally accepted as an important medium for the calcification process. While the role this “calcified organic matrix” plays in the calcification process has long been appreciated, the complex mixture of proteins that is induced and assembled during the mineral phase of calcification remains uncharacterized in many organisms. Thus, we investigated organic matrices from the calcitic sclerites of a soft coral, *Sinularia sp.,* and used a proteomic approach to identify the functional matrix proteins that might be involved in the biocalcification process. We purified eight organic matrix proteins and performed in-gel digestion using trypsin. The tryptic peptides were separated by nano-liquid chromatography (nano-LC) and analyzed by tandem mass spectrometry (MS/MS) using a matrix-assisted laser desorption/ionization (MALDI) – time-of-flight-time-of-flight (TOF-TOF) mass spectrometer. Periodic acid Schiff staining of an SDS-PAGE gel indicated that four proteins were glycosylated. We identified several proteins, including a form of actin, from which we identified a total of 183 potential peptides. Our findings suggest that many of those peptides may contribute to biocalcification in soft corals.

## Introduction

Soft corals contain small spicules of CaCO_3_ called “sclerites”, which are biomineralized structures composed of an organic matrix and a mineral fraction [1], [2], [3]. They define key parameters for the structural design of a soft body [3]. The formation of biominerals by living organisms is fastidiously controlled by proteins and polysaccharides [4], [5] that ensure the development of crystals with a particular composition and morphology, guarantee the manufacture of structures with strictly defined shapes, and dictate the internal and external physicochemical properties of the resulting materials [5], [6], [7]. Prominent among these are intracrystalline proteins, which are distributed throughout the individual biomineral crystals and play a vital role in dictating the texture and physical properties of calcified tissues of most marine organisms [6]. The organic matrix associated with the sclerites of soft corals primarily consists of proteins. Therefore, to better understand the calcification process of endoskeletal sclerites in marine organisms, functional biomolecules should be identified. We applied a proteomic approach, one of the best ways to identify functional molecules, to identify the proteins thought to be involved in the biocalcification of endoskeletal sclerites and to gain a more complete understanding of the calcification process.

We purified organic matrix proteins from the sclerites of a soft coral, *Sinularia sp.,* and performed in-gel digestion using trypsin. The tryptic peptides were separated by a nano-LC and analyzed by MS/MS using a highly powerful MALDI-TOF-TOF mass spectrometer. Although marine organisms have been reported to contain carbonic anhydrase [8], [9], [10], [11], [12], calcium-binding and glycosylated proteins [1], [10], [13], [14], this is the first report of the identification of actin in sclerites. Actin is a protein that has not previously been shown to play a role in the biocalcification process of other corals except in the planular larvae of the scleractinian *Galaxea fascicularis* [15], but it has been suggested that actins reach the area of biomineralization as a by-product of the secretion of other proteins involved in calcification and may not be directly involved in the biomineralization process [17]. Our results cannot decide so far whether soft coral sclerites might be formed through a biological process using the actin protein, similar to other biomolecules that were already reported in this group.

Actin is a protein that functions with myosin in muscle contractions [16]. It helps to construct and maintain the cytoskeletal structure within cells and forms microfilaments in mammalian cells. Actin also contributes to biological processes such as sensing the environment, internalizing membrane vesicles, moving over surfaces, and cell division. These cellular activities are complex; they depend on the interactions of actin monomers and filaments with numerous other proteins. As such, actin is a central player in cell shape and movement and participates in many important cellular processes including muscle contraction, cell motility, cell division and cytokinesis, vesicle and organelle movement, cell signaling, and the establishment and maintenance of cell junctions and cell shape [16]. An important, unresolved question is whether actin has a function in the incorporation of extracellular materials during the calcification process in marine organisms. Recently, actin was identified in the intracellular organic matrix of sea urchin tests and spines [17] and in the planular larvae of scleractinian corals [15] but its direct involvement in the biocalcification process has yet to be demonstrated. Here, we found that actin proteins in the sclerites of *Sinularia* sp., which also contain the mineral calcite [14], [18], are secreted together with an organic matrix and subsequently transported to the outside of the cell where extracellular calcification occurs. This process is similar to sclerite calcification in other octocorallians [19], [20]. Mature sclerites are completely free of cellular materials and ultimately become extracellular structures. We are tempted to exclude actin contamination from the surrounding cells as an explanation for this finding due to the many peptide fragments identified as actin from a complex protein mixture that was purified from silver-stained protein bands using our newly established matrix protein purification technique [1], [14]. However, verification of these proteins using a number of techniques (e.g., CBB staining, western blotting, silver staining, and analyses by nanoLC-MS/MS using a MALDI-TOF-TOF) was done several times on the same protein bands with the same results.

The sensitivity and high-throughput nature of modern mass spectrometry (MALDI-TOF-TOF) enables the implementation of a proteomic approach to explore the entire protein set of a complex mixture at once. Consequently, we used such an approach for an overview analysis of the matrix proteins contained in the soft coral *Sinularia sp*. The total soluble organic matrix (SOM) and the eight main purified proteins (dubbed SSCL-150, SSCL-100, SSCL-75, SSCL-63, SSCL-42, SSCL-33.5, SSCL-31, and SSCL-14 according to their predicted molecular weights) were investigated. Approximately 180 internal peptide sequences were determined by de novo sequencing. The sequence analysis strongly suggested that the protein migrating at approximately 150 kDa (i.e., SSCL-150) potentially played a role in the sclerite calcification process. Additionally, the peptide sequences corresponding to the SSCL-75 and the SSCL-14 bands matched proteins annotated as thioredoxin, hypothetical and fluorescent proteins.

## Results and Discussion

### Calcified Endoskeletons (Sclerites)

**Figure 1:**
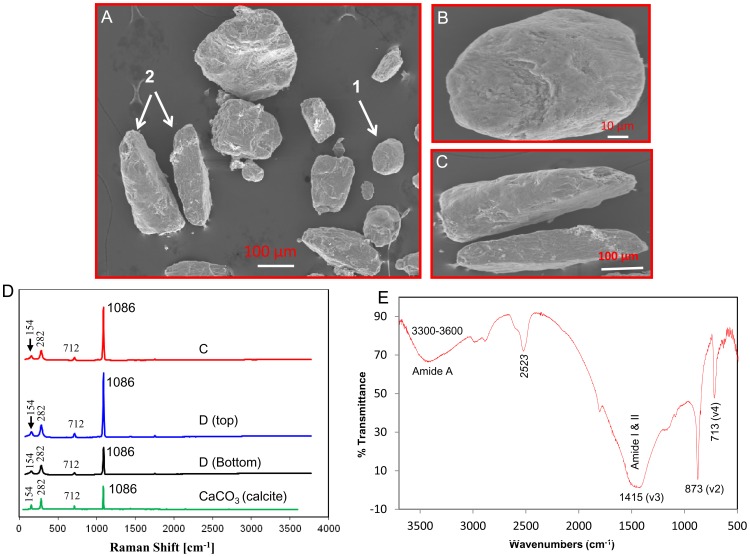
(A) SEM images of mature sclerites isolated from the colonies of *Sinularia sp*. Two types of sclerites were identified; arrows 1 and 2 indicate egg-shaped and rod-like sclerites, respectively. (B) Enlarged view of an egg-shaped sclerite. (C) Enlarged view of rod-like sclerites. (D) Raman spectra with the different sclerite shapes (Fig. B and C show the enlarged view). Characteristic Raman bands on both “egg-shaped” (top) and “rod-like” (second and third from the top) sclerites showed calcitic crystals at 154, 282, 712 and 1086 cm^−1^. Several points in each sclerite were used for Raman analysis with similar results for each point. (E) FTIR spectra of the powdered sclerite samples showing the various molecular conformations, pure mineral composition and diagnostic IR absorption frequencies for proteins and amide A. Three strong calcite bands were identified at 713 cm^–1^ (v4), 873 cm^–1^ (v2) and 1415 cm^–1^ (v3). Structural proteins of amide A (3300–3600 cm^–1^), amide I & II (1400–1800 cm^–1^), and a triple bond (2523 cm^–1^) were also identified.

The sclerites of *Sinularia sp.,* when observed under a scanning electron microscope (SEM), appeared irregularly shaped; some were rod-like, and others were egg-shaped. The sclerites varied in size from approximately 100 to 250 µm. (Fig. 1). These sclerite shapes are quite different from a previously identified specimen from the same family, Alcyoniidae [1], [3], [13], [21]. Although frequently detected in other soft coral sclerites [1], [13], spurs were not observed in the sclerites of *Sinularia sp.* (Fig. 1B). Because the shape of these sclerites were completely different from other soft coral sclerites [1], [3], [13], [14], [20], we investigated sclerites representing each of the different shapes to determine whether they indeed induced calcite mineralogy as do sclerites in other soft corals. To confirm the mineral composition, we performed micro-Raman spectroscopy on the different shapes and FTIR analysis of the powdered samples of the sclerites. The results of both analyses clearly indicated calcitic mineralogy (Fig. 1D, E; see figure legend for details).

Sclerites are usually found in the axis as well as the cortex of a colonial skeleton, although they may also be present in the tentacles, pharynx and upper part of the autozoids [22]. Notably, sclerites are found in the subclass Octocorallia, including members of the Gorgonacea and Alcyonacea; all sclerites in this subclass are composed of calcite [14], [20]. We noted that the sclerite shape in *Sinularia sp.* was different from that of other Alcyonacea [3], [23] and Gorgonacea [24]. Scale-shaped or rounded sclerites with a spherulitic structure were formed by gorgonians while spindles and rod-shaped spicules were common among gorgonians and pennatulids. These observations clearly suggest that sclerite shapes are species-specific and that within a species they generally differ according to the anatomical site at which they are formed. Very recently, a crystallographic structure [25] and a distribution pattern of calcite mineral [26] in soft coral sclerites have been reported in detail. However, although morphological studies of sclerites have been previously reported and form the basis of octocoral taxonomy, the complete biochemical and proteomic analyses of these important skeletal elements has been lacking to date. Here, we present the results of such analyses.

### Proteinaceous Organic Matrices

**Figure 2:**
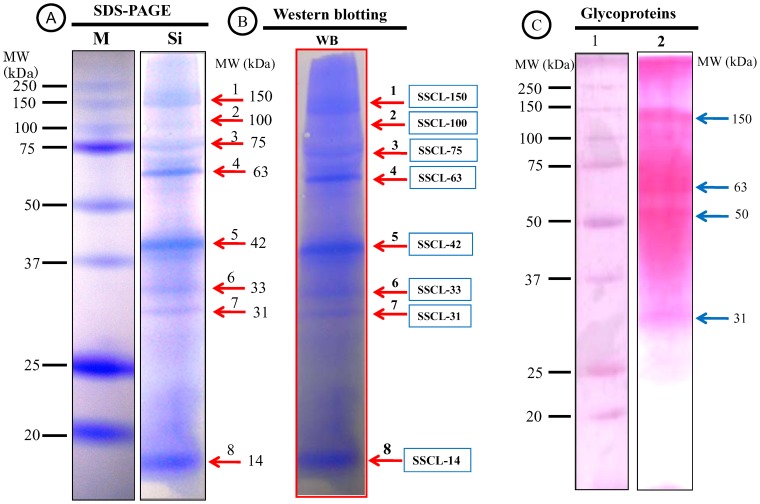
Electrophoretic analysis of matrix proteins extracted from the calcitic sclerites. (A) SDS-PAGE fractionation with CBB staining after purification of the proteins. An eluate (derived from 10 g of sclerites) was run on a 12% polyacrylamide gel. M, protein marker; Si, purified proteins of *Sinularia sp.* The arrows indicate protein bands. (B) Western blotting (WB) of matrix proteins on a PVDF membrane; proteins correspond to those on the SDS–PAGE gel shown in A. The arrows indicate same purified proteins with the same numbers (1–8) as shown in A. The eight purified proteins were named according to their origin (*Sinularia sp*. sclerite; SSCL) and their apparent molecular mass as follows: SSCL-150, SSCL-100, SSCL-75, SSCL-63, SSCL-42, SSCL-33.5, SSCL-31, and SSCL-14. (C) SDS-PAGE gel with PAS staining to identify the glycoproteins in the soluble organic components of sclerites. An eluate (derived from 10 g of sclerites) was run on a 12% polyacrylamide gel. *Lane 1*, protein marker; *Lane 2,* four glycoproteins were identified (indicated by arrows) by periodic acid-Schiff staining.

After decalcification of calcitic sclerites, the SOM comprised 0.04% of the sclerite weight and emerged as a white fluffy material. SDS-PAGE (12%) analysis of the decalcified samples revealed eight bands of proteins with apparent molecular masses of 150, 100, 75, 63, 42, 33.5, 31 and 14 kDa. The electrophoresis pattern of SOM stained with CBB, alongside standard proteins of known molecular weights, is shown in Figure 2A. Spectrometric analysis indicated that partial sequences of many different proteins were present in the stained bands, suggesting that these bands corresponded to a mixture of proteins rather than a single protein.

Among these bands, five proteins with apparent molecular masses of 150, 75, 63, 42 and 14 kDa were the most abundant (Fig. 2A, Lane Si). An additional band at 100 kDa was very weak. To confirm the purification, the proteins on the SDS–PAGE were transferred to a polyvinylidene difluoride (PVDF) membrane using a western blot technique (Fig. 2B, WB). To identify any glycosylated proteins, a new SDS-PAGE was subsequently performed under the same experimental conditions as those described above. After staining the SDS-PAGE gel with PAS stain, four of the eight dominant sclerite proteins (SSCL-150, SSCL-63, SSCL-50, SSCL-31) were shown to be glycosylated (Fig. 2C, Lane 2).

These results revealed that the proteinaceous organic matrices obtained from the sclerites of *Sinularia sp.* were more abundant than those of the closely related *Lobophytum crassum*, for example [13]. PAS staining indicated that four major bands of proteins contained glycoproteins (Fig. 2C, Lane 2). Glycoproteins are known to function as inhibitors or stabilizers by binding the precursor amorphous CaCO_3_ (ACC), which acts as a template for the transition of ACC to structured crystals on an insoluble or soluble organic matrix [27]. Of the eight proteins isolated, four showed strong glycosylation, revealing the potential function of these proteinaceous matrices. In addition, actin and thioredoxin sequences were obtained from glycosylated protein bands indicating that these two important proteins are glycosylated. Glycoproteinaceous matrices are commonly found in other calcifying systems [28]. Tompa et al. [29] described the insoluble matrix of snail egg shells as being a sulfated glycoprotein of 53 kDa. Based on studies of proteins in other animals, it appears that actin is also glycosylated [30]. However, this is the first study to identify actin as one of the glycosylated proteins found in the organic matrix of a calcifying marine organism. As mentioned above, glycosylated proteins are important for the biomineralization process. Evidence from octocorallians and from our previous reports [1], [13], [14] suggest that at least one protein involved in biomineralization in calcifying marine organisms must be a glycoprotein. Therefore, the functional role(s) of glycosylated proteins in calcifying organisms, and in particular within the octocorallians, definitely requires further investigation. The presence of an organic matrix or sheath appears to be a requirement for all calcifying systems. Kingsley et al. [28] reported that mature sclerites contained 5.9% organic material by weight, most of which was protein; an observation supported by our results. Gel electrophoresis revealed the presence of many proteins along with their amino acid compositions. It has been reported that protein is always present in scleractinian coral skeletons [31] and that it comprises approximately 0.3% of the dry skeleton by weight [31], [32].

### Proteomic Analyses

**Figure 3:**
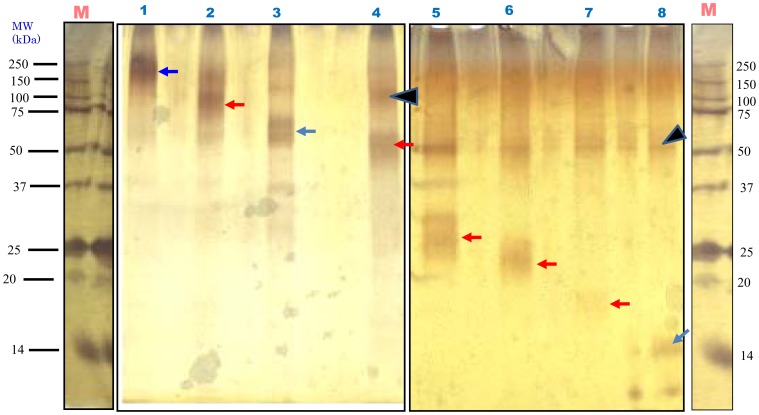
Silver staining of purified matrix proteins. *Lanes 1–8,* eight bands of proteins with the apparent molecular masses of 150 (SSCL-150), 100 (SSCL-100), 75 (SSCL-75), 63 (SSCL-63), 42 (SSCL-42), 33 (SSCL-33), 31 (SSCL-31) and 14 (SSCL-14) kDa, respectively. M, protein marker. Each arrow indicates a protein band. Arrowheads indicate constant contaminants of control samples by SDS-PAGE. This contamination did not interfere with the analysis of the proteins. Blue arrows indicate the proteins that were subjected to in-gel trypsin digestion for identification by MALDI-TOF-TOF analysis.

**Figure 4:**
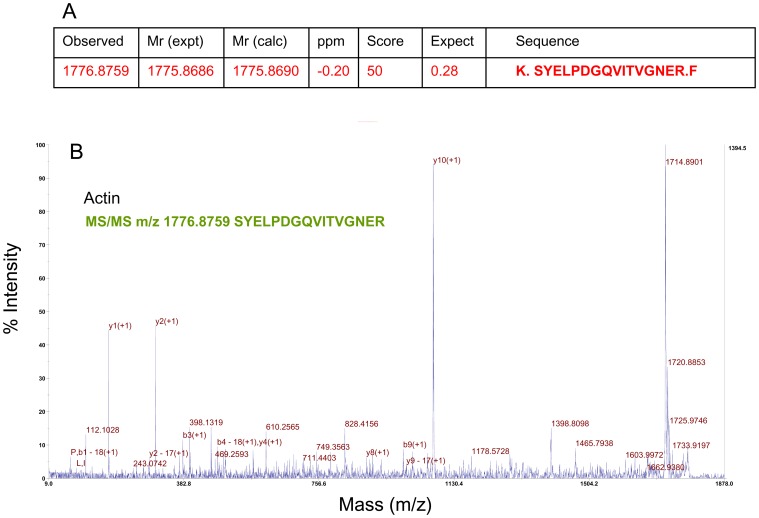
(A) MS/MS data from a precursor peptide of SSCL-150 with an *m/z* ratio of 1776.87 after trypsin hydrolysis. (B) Typical MS/MS spectrum for actin acquired using MALDI-TOF-TOF. See Tables 1 and S1 for details.

To identify the proteins precisely, we visualized all purified proteins by silver staining (Fig. 3) followed by digestion with trypsin. The peptide sequences determined by the *de novo* interpretation of MS/MS spectra are shown in Table 1 for the main protein band SSCL-150. The peptide sequences shown in Table 1 were identified with 90–99.9% confidence. Those with the closest homology were identified from both the NCBI and the Anthozoa databases. An example of an MS/MS spectrum for actin is shown in Figure 4. A screenshot for actin obtained from ProteinPilot™ is shown in Figure S1 with the fragment peaks numbered and labeled; the spectra, with parent ions indicated by a red arrow, are shown in Figure S2. The MS/MS spectra for this protein revealed that the main protein identified in *Sinularia sp.* was actin. In total, we obtained approximately 183 actin peptide fragments from the SSCL-150 band (Table S1).

**Table 1:** *De novo* peptide sequences obtained after the tryptic digestion of purified soluble matrix proteins from silver-stained SSCL-150.

Identified proteins	m/z	Sequences	Accession No. |gi|
Actin	1776.875	SYELPDGQVITVGNER	gi|67462785;gi|167376754;gi|158914;gi|163716525
Actin	1198.727	AVFPSIVGRPR	gi|67462785;gi|167376754;gi|158914
predicted	1302.742	SLDLDSIIAEVK	gi|189054178;gi|160961491;gi|1346343;gi|119395750;gi|11935049
Actin	1499.818	EMIQLAPPTMKIK	gi|67462785;gi|167376754;gi|158914
Actin	1782.845	MGQKDAYVGDEAQSKR	gi|67462785;gi|167376754;gi|158914
Actin	1083.631	AILRLDLAGR	gi|38176182
Predicted	1251.589	STVIDKCSANR	gi|156397283;gi|156224936
Predicted	1232.567	MFCAGYLQGGK	gi|91087943;gi|559508
hypothetical protein	1707.906	ENEAGEVDAEAVRYR	gi|154343676
hypothetical protein	1708.899	ENEAGEVDAEAVRYR	gi|154343676
hypothetical protein	1769.826	ENEAGEVDAEAVRYR	gi|154343676
hypothetical protein	1507.847	GGRVNVANPRTPGGR	gi|116191187
azurin precursor	1483.688	EVIAAANAKLADCR	gi|32472058
class II aldolase/adducin family protein	1488.779	GAVVSGEGRASTELR	gi|153003236
Protocadherin gamma-B2 precursor	1890.914	EDVPPGFFVLQVTATDR	gi|62510862;gi|14270493;gi|127138947;gi|114602349;gi|11128035;gi|109079000
glycyl-tRNA synthetase subunit beta	1187.588	SIQSYADFQK	gi|56963450
hypothetical protein	2098.955	DASWPGDNAPSEMLPNEIR	gi|54295960
apocytochrome c	1168.639	TGPNLHGLFGR	gi|914118;gi|914117;gi|89272116;gi|82408001;gi|75056683;gi|74422779;gi|6681095;gi|57096040
ApbE-like lipoproteinABC Transporter	1609.73	AWGFGSEPANAEAMR	gi|69937557
substrate-binding protein	1027.584	GVGKILIDGR	gi|36959117;gi|13475809
thioester reductase domain containing protein	1197.626	GERVAILLGNR	gi|194339623
seryl-tRNA synthetase	1743.872	EGKNAEAEALIEEGKR	gi|170289239
methyl-accepting chemotaxis protein	1199.718	GVVGGPNAAASSGR	gi|76810025;gi|67643497;gi|53724505;gi|194511256;gi|194504629;gi|121601458
Predicted	1684.793	RLFVDQELPDIPSR	gi|73975422
Protein apaG; ApaG	1109.603	WTITDGFNR	gi|50400623;gi|16126478
Amino acid adenylation domain-containing protein	2258.068	RIYTLQQMEGIGTSYNMPR	gi|163939762
Predicted	2146.264	ARTSLLPVGGGALLYAPPAPPK	gi|126310787
EIF4G1 variant protein	707.3291	GSSGGSGAK	gi|68533081;gi|57997536;gi|41019505;gi|3941724;gi|38201621
hypothetical protein CHGG_08558	1840.915	TLLYQIVQQAPEIAPR	gi|116201347
two-component sensor kinase CbrA	1843.816	LELDHSAHQAHTQSIR	gi|114319836
hypothetical protein CAGL0I02860g	1136.571	LGSTSSVNLMK	gi|50289887
metallophosphoesterase	1485.716	GVSVRLMQPVIDR	gi|91775253
pyridine nucleotide-disulfide oxidoreductase family protein	1111.583	MKGYPASVMK	gi|167015501
hypothetical protein PVX_113390	1320.608	SSLMNVSRWLK	gi|156100753
Predicted: similar to cytochrome P450 2P11	1976.98	EFRPERFLDSEGNPKR	gi|72023628;gi|115725228;gi|115642010
hypothetical protein	1114.624	IAHWFWVR	gi|118579186
hypothetical proteinFG04875.1	1755.082	AAVASDPSIQVLALPFR	gi|46120456
DNA binding	1358.672	GDDQLNGLQGGKR	gi|15225125

Sequences were deduced from MS/MS spectra. The peptide sequences were generated with 90–99.9% confidence, and the best matches were identified from both the NCBI and the Anthozoa databases.

**Figure 5:**
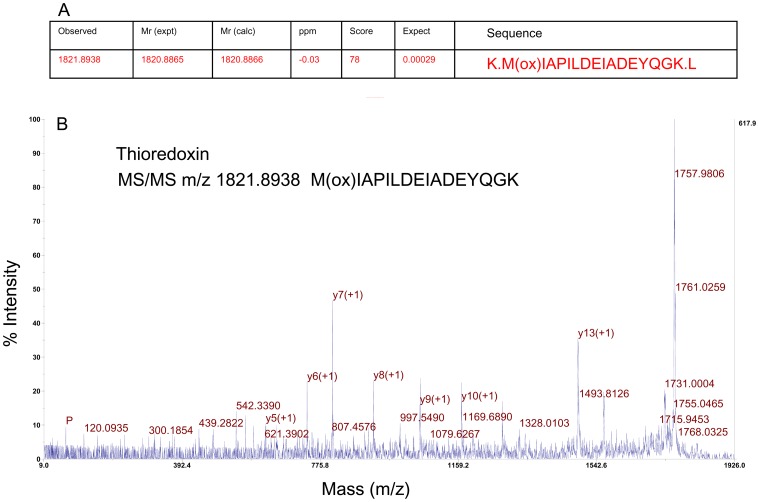
(A) MS/MS data for the peptide precursor with an *m/z* ratio of 1739.86 from the SSCL-75 tryptic digest. (B) Typical MS/MS spectrum for thioredoxin acquired using MALDI-TOF-TOF.

We also identified a mixture of thioredoxin (nine peptides) and several chain (A, E) proteins from the SSCL-75 band (Table 2). The MS/MS spectra of a thioredoxin peptide (*m/z* = 1821.89) is shown in Figure 5. A screenshot for thioredoxin obtained from ProteinPilot™ is shown in Figure S3 with the fragment peaks numbered and labelled; the spectra, with parent ions indicated by a red arrow, are shown in Figure S4. This study is the first to identify thioredoxin, which usually functions as an antioxidant, in soft coral sclerites. However, SSCL-75 was not identified as a glycoprotein by periodic acid Schiff analysis, suggesting that this protein has a limited function in the biocalcification process. Some other proteins, such as hypothetical and fluorescent, might have functional roles in biocalcification [33], [34], [35], although the putative function of the hypothetical proteins could not be defined.

**Table 2:** A list of proteins identified by MS/MS spectra from silver-stained SSCL-75.

Identified proteins	Peptide matches	Accesion No. |gi|
**Thioredoxin**	1	gi|84393642
**Thioredoxin**	1	gi|26250519
**Thioredoxin/transketolase fusion protein**	1	gi|25067747
**Thioredoxin**	1	gi|16762214
**Thioredoxin**	1	gi|161505567
**Thioredoxin**	1	gi|15804371
**Thioredoxin**	1	gi|156935888
**Thioredoxin**	1	gi|148071
**Thioredoxin**	1	gi|146313636

The proteins were obtained with 80–90% confidence, and the best matches were identified from the NCBI database.

In this study, we used a combination of biochemistry and proteomics to characterize the organic matrices of sclerites in the soft coral *Sinularia sp*. Our results revealed that the organic matrix of these sclerites was composed of eight principal, discrete, proteinaceous fractions, including four glycoproteins, several predicted proteins, and thioredoxin, which may play a role in the biocalcification of the sclerites. During our database mining, we noted that sequences indicative of actin were associated with a significant number of diverse species (Table 1 and S1) implying that this protein in soft corals is homologous with that of many other animals. These partial sequences match not only with known proteins from the NCBI database but also with unknown proteins because the transcriptome/genome of *Sinularia sp*. is not currently available; these partial sequences could be part of another protein. To date, a few actin-binding proteins were identified in marine organisms [36], [37], [38], including a high molecular weight (250 kDa) actin-binding protein from a sea urchin egg cytoplasmic extract [36]. In another report, Mann et al. [17] noted that actin was present in the test and spine matrices of sea urchins. However, in this report, the authors hypothesized that this protein may have reached the mineralization space as by-products of the secretion of specific matrix proteins or of mineral precursors, or alternatively were may have been derived from damaged cells of the cell layer confining the mineralization space.

Many different proteins which modify actin polymerization are thought to regulate actin assembly and the formation of the spectacular variety of actin structures [37], [39], [40], [41], [42], [43], [44]. The effort to determine how assembly and interconversion of the diverse polymeric forms of actin are regulated has led to the discovery of many actin-binding proteins [36], [45], [46], [47]. There appears to be no limitation to the ranges of molecular weight of actin-binding proteins, because many high molecular weight (90–270 kDa) actin-binding proteins have already been identified in diverse species [30], [36], [42], [48], [49], [50], including marine organisms [36]. These findings are consistent with the present report.

There is a great abundance of *Sinularia sp.* in Okinawa, but organisms have not yet been identified at the species level because of a lack of primary information. It might be possible to determine the species by mitochondrial RNA/DNA studies, and we believe that the primary sequences obtained here will be a useful first step for identifying species of these abundant organisms. Combining proteomics with transcriptomics data could be helpful for such work. In addition, these results might be useful for further studies in which the genes that encode these proteins could be amplified using RACE experiments to isolate the full-length DNA sequences and, in turn, the complete amino acid sequences of the detected proteins. Additionally, the results of this study could be used for designing commercial antibodies against the actin protein to label and locate it within the organic matrix of the sclerites. Overall, the present study is preliminary; however, the peptide sequences obtained here may serve as a road map for further studies dealing with organic matrix and calcification.

Except for a few reports on mollusk shells [9], [10], [51], [52], [53] and sea urchins [17], [54], [55], few proteomics analyses have been performed on calcifying marine organisms. In particular, the protein composition of the organic matrix of scleractinian and octocoral biominerals is poorly characterized. Our present data now contribute to closing this gap thus enabling a broader comparative proteomic approach for studying organic matrices in marine organisms.

## Experimental Section

### Specimen Collection

Colonies of *Sinularia sp.* were collected by scuba diving to depths ranging from 10 to 20 meters at low tide near the Sunabe coast of Chatan, Okinawa, Japan**.** No specific permits were required for the described field studies. The field studies did not involve endangered or protected species.

The collected colonies were vigorously washed with tap water in the laboratory to remove excess salts and other unwanted substances. The colonies were then cut into small pieces using sharp scissors, washed with tap water again, and stored at −20°C until use.

### Isolation of Endoskeletons

Calcified endoskeletons (sclerites) were isolated from each coral colony (*Sinularia sp.*) according to the series of mechanical and chemical treatments described by Rahman et al. [14]. Briefly, pieces of coral colony were ground five to six times in a mixer machine (BM-FE08, Zojirushi, Japan) and washed with tap water until the sclerites were obtained. The collected sclerites were cleaned by stirring in 1 M NaOH for 2 h followed by vigorous stirring in a 10% sodium hypochlorite (NaOCl) bleaching solution for 30 min to remove fleshy tissues and debris; following these washes, the sclerites were considered to be completely tissue-free. The treated samples were washed under tap water to remove the cleaning solutions. Finally, the samples were washed with MilliQ water five times to remove any additional contaminants. The sclerites were examined using a microscope (Nikon ECLIPSE E 200, Japan) to determine whether they were completely free of tissues and other contaminants.

### Scanning Electron Microscopy (SEM)

The completely cleaned mature sclerites were subjected to observation under an SEM. Mechanically and chemically treated sclerites were examined for the shape and effect of chemicals during the separation of sclerites from the soft tissues. All specimens were placed in a sample holder, dried for a few days, and coated with palladium gold using an ion coater (E-1010, Hitachi, Japan). The samples were examined under an SEM (Hitachi S-3000 N, Japan) operated at 15 kV.

### Extraction of Proteinaceous Organic Matrices from Sclerites

To extract the proteinaceous organic matrix components from the sclerites, we used the protocol described in our previous works [1], [14]. The mechanically and chemically cleaned (see method for isolation of endoskeletons above) sclerites were decalcified in 0.5 M ethylenediaminetetraacetic acid (EDTA)-4 Na (pH 7.8), overnight at room temperature. The decalcifying solution was centrifuged (H-103, Kokusan) at 4000 rpm for 20 min to remove any insoluble materials and was then passed through filter paper (Whatman). To remove the EDTA, the solution was dialyzed against 5×1 L of ultrapure water (MilliQ) for 64 h using a dialysis membrane (UC36-32-100, Viskase Companies, Inc., Japan). The water was changed five times during this period.

### Purification of Matrix Proteins

We purified proteins from the sclerites according to the series of methods described by Rahman et al. [14]. Briefly, the matrix components that had been extracted as described above were prepared for electrophoresis analysis as follows:

1. The filtered samples were passed in tandem through two Sep-Pak® Plus C_18_ cartridges (Waters) containing a silica-based bonded phase with strong hydrophobicity that typically adsorbs weakly hydrophobic components of aqueous solutions. This behavior is similar to that of reverse-phase HPLC columns. The samples were injected using a peristaltic pump (NeoLab Mini Pump) at a flow rate of 1 drop/s to separate the soluble macromolecules. The Sep-Pak® Plus C_18_ cartridges were activated by methanol (4 mL/3 times) prior to the application of the samples. The cartridges were washed with distilled water (DW) (6 mL/2 times) after activation. After passing the samples through the cartridges, most of the EDTA was assumed to have been removed, and the macromolecules were assumed to remain in the cartridges. To completely remove the EDTA, we passed DW (6 mL/5 times) through each cartridge followed by 10% acetonitrile (2 mL/3 times). Finally, the absorbed macromolecules were eluted in 50% acetonitrile (2 mL/3 times). The eluted macromolecules were frozen at −80°C and subsequently lyophilized.
2. Each lyophilized sample was weighed (2 mg) and mixed with 0.4 mL of 99% chloroform (CHCl_3_), 0.2 ml of 100% methanol (MeOH), and 0.4 ml of H_2_O, and then centrifuged (1500 rpm) using an IKA® VORTEX (GENIUS 3) three times for six min each to remove the lipids and other unwanted substances. The sample was then concentrated by centrifugation in an Amicon Ultrafree-MC (Millipore) column using a Minispin Plus centrifuge (Eppendorf) and dried using a centrifugal concentrator (VC-36N, TAITEC) under a vacuum at 60°C. At this point, the sample was ready for gel electrophoresis.
3. Each dried, purified matrix protein sample was dissolved in Laemmli sample buffer (Bio-Rad) at a ratio of 2∶1 (sample buffer to protein sample) and denatured at 100°C for three min. The samples were then subjected to 12% sodium dodecyl sulfate-polyacrylamide gel electrophoresis (SDS-PAGE) [56] for approximately 1 h. The Precision Plus SDS-PAGE standard (Bio-Rad) was used as the protein marker for the electrophoresis. After electrophoresis, the gel was stained with Coomassie Brilliant Blue (CBB) (1% CBB R-250 in 40% MeOH and 7.5% acetic acid) and destained (10% MeOH and 7.5% acetic acid) to visualize the protein bands. The purification of proteins was further checked by silver staining and western blotting.

### Western Blotting on PVDF Membranes

The purified sample underwent electrophoresis with SDS-PAGE using the same technique as described above. After undergoing electrophoresis, the gel was kept in 100% MeOH for 15 s and shaken in buffer for 10 min. Subsequently, the Mini Trans Blot^(R)^ (Bio-Rad) was used to transfer the sample onto a polyvinylidene difluoride (PVDF) clear membrane at 350 mA and 60 V for 4 h. The membrane was stained with CBB (1% CBB, 50% MeOH) and destained (50% MeOH, 5% acetic acid) for visualization of the bands; the membrane was then washed with distilled water and dried overnight.

### Periodic Acid Schiff (PAS) Staining

PAS staining was used in the SDS-PAGE gel to identify glycoproteins. This staining was conducted using the Schiff (fuchsin-sulfite) reagent according to the method developed by Segrest et al. [57]. A strong red/purple staining of a band indicated the presence of a glycoprotein.

### Protein Digestion and MALDI-TOF-TOF Analysis

After electrophoresis and CBB (R-250) staining, we excised all protein bands from the gel and performed electroelution treatments for further purification of the proteins, following protocols described in our previous work [1]. After electroelution treatment, we performed SDS-PAGE again and subsequently visualized all purified proteins by silver staining (Bio-Rad product). The silver-stained purified proteins were excised from the gel and cut into smaller pieces prior to further processing. The gel pieces were washed with 50% 50 mM ammonia carbonate buffer (pH 8.0), 50% acetonitrile, and pure acetonitrile, in sequence. After the final wash, the gel pieces were dried under a vacuum. The cysteines were reduced with dithiothreitol **(**DTT) and alkylated with iodoacetamide. The proteins were digested in-gel with trypsin (Promega; Madison USA) overnight, and the gel pieces were extracted with 50% acetonitrile in 0.1% trifluoroacetic acid (TFA).

The nano-LC (Dionex RSLC, Sunnyvale, USA) was set up with solvents A (95% H_2_O, 5% acetonitrile, 0.1% TFA) and B (80% acetonitrile, 20% H_2_O, 0.1% TFA). A spotter (Dionex Probot, Sunnyvale, USA) was set up with a matrix consisting of 2 mg/ml α-Cyano-4-hydroxycinnamic acid (CHCA) in 70% acetonitrile with 0.1% TFA and a fraction size of 12 min/s. The peptide samples were separated using the following gradient: 0 to 5 min, 5% solvent B; 5 to 65 min, 5%–50% solvent B; and 65 to 66 min, 50%–95% solvent B.

The peptides separated by nano-LC were analyzed in MS reflector mode. The mass range for MS acquisition was 700 to 4000, and 2000 shots were summed for one spectrum. Precursors for MS/MS fragmentation were selected automatically, selecting a maximum of 20 precursors per fraction with a minimum signal to noise ratio of 50. MS/MS spectra were smoothed using the Savitzky-Golay filter method [58] before interrogating the databases. MS and subsequent MS/MS analyses were performed using the AB SCIEX TOF/TOF™ 5800 System (AB SCIEX, Foster City, USA).

### Mass Spectrometry and Database Searching

All MS/MS spectra were analyzed using the following databases: (1) Mascot® using the NCBI database (NCBI 2010) with all entries, (2) Mascot® using the sub-database Anthozoa (extracted from NCBI), (3) ProteinPilot™ 3.0 Software (AB SCIEX, Foster City, USA) using the NCBI database with all entries, (4) ProteinPilot™ using the sub-database Anthozoa (extracted from NCBI), (5) *de novo* sequencing using the Denovo Explorer™ and a BLAST search against NCBI 2010, and (6) *de novo* sequencing using the Denovo Explorer™ and a BLAST search against the Anthozoa database.

For the MS/MS database search, the MS/MS data (i.e., fragment masses) were compared against theoretical fragment masses calculated from all protein sequences in the database. For the *de novo* sequencing analysis, the MS/MS spectra were used to create a sequence tag (i.e., the mass difference between fragment peaks in the MS/MS spectra could be explained by differences in amino acid composition). The amino acid sequences were then compared against the protein database.

The number of coral proteins in the database is limited. The ProteinPilot™ software using the Paragon algorithm [59] offers the possibility of searching for amino acid substitutions, which increases the chance of finding proteins by homology. In addition, the Mascot search engine was used to find further evidence to confirm the identity of the peptides. In the current circumstances, where proteins are identified based on a low number of peptides due to poorly populated databases, identification based on two different search algorithms provides a higher confidence than if only one algorithm was considered.

## Supporting Information

Figure S1 **Screenshot of actin peptide obtained from ProteinPilotTM.**
(PDF)

Figure S2 **The parent ion of actin.** (A, B) The parent ion on the spectra (red arrows).
(PDF)

Figure S3 **Screenshot of Thioredoxin peptide from ProteinPilotTM.**
(PDF)

Figure S4 **The parent ion on the spectra of Thioredoxin (red arrow).**
(PDF)

Table S1 **A list of proteins identified by** ***de novo*** **analysis from silver-stained SSCL-150 and highly conserved with other animals proteins.** The proteins were obtained with 80–95% confidence, and the best hits were from the NCBI databases. All identifications were made by tandem MS/MS searches.
(PDF)
