# Correction: Analysis of the Proteinaceous Components of the Organic Matrix of Calcitic Sclerites from the Soft Coral *Sinularia sp.*

**DOI:** 10.1371/annotation/40505aa8-17cf-49c0-8d1d-1e28af8553cb

**Published:** 2013-11-14

**Authors:** M. Azizur Rahman, Ryuichi Shinjo, Tamotsu Oomori, Gert Wörheide

In Figure 1 (D), the spectra line labels C, D (top), and D (Bottom) are incorrect. The correct figure can be viewed here: 

**Figure:**
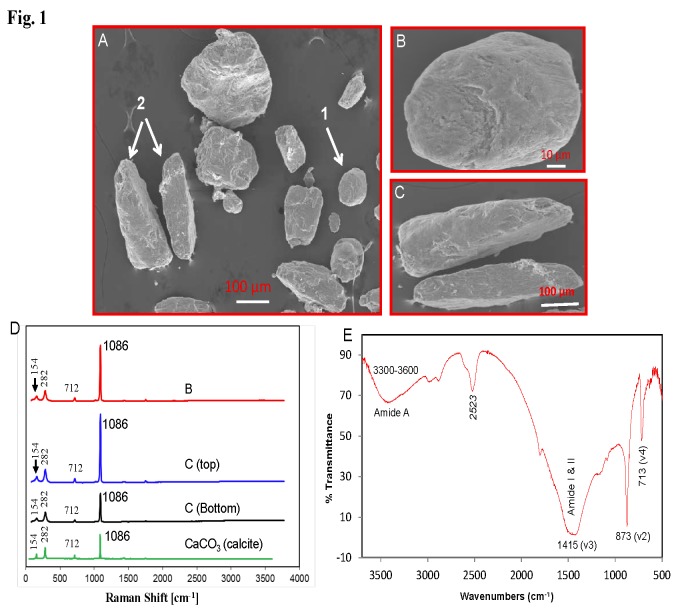


f

