# Supplementary figures and images for: Analysis of the Proteinaceous Components of the Organic Matrix of Calcitic Sclerites from the Soft Coral Sinularia sp
Source: PLoS One. 2013 Mar 14;8(3):e58781. doi: 10.1371/journal.pone.0058781 (PMC3597568; doi:10.1371/journal.pone.0058781)

Fig. S1

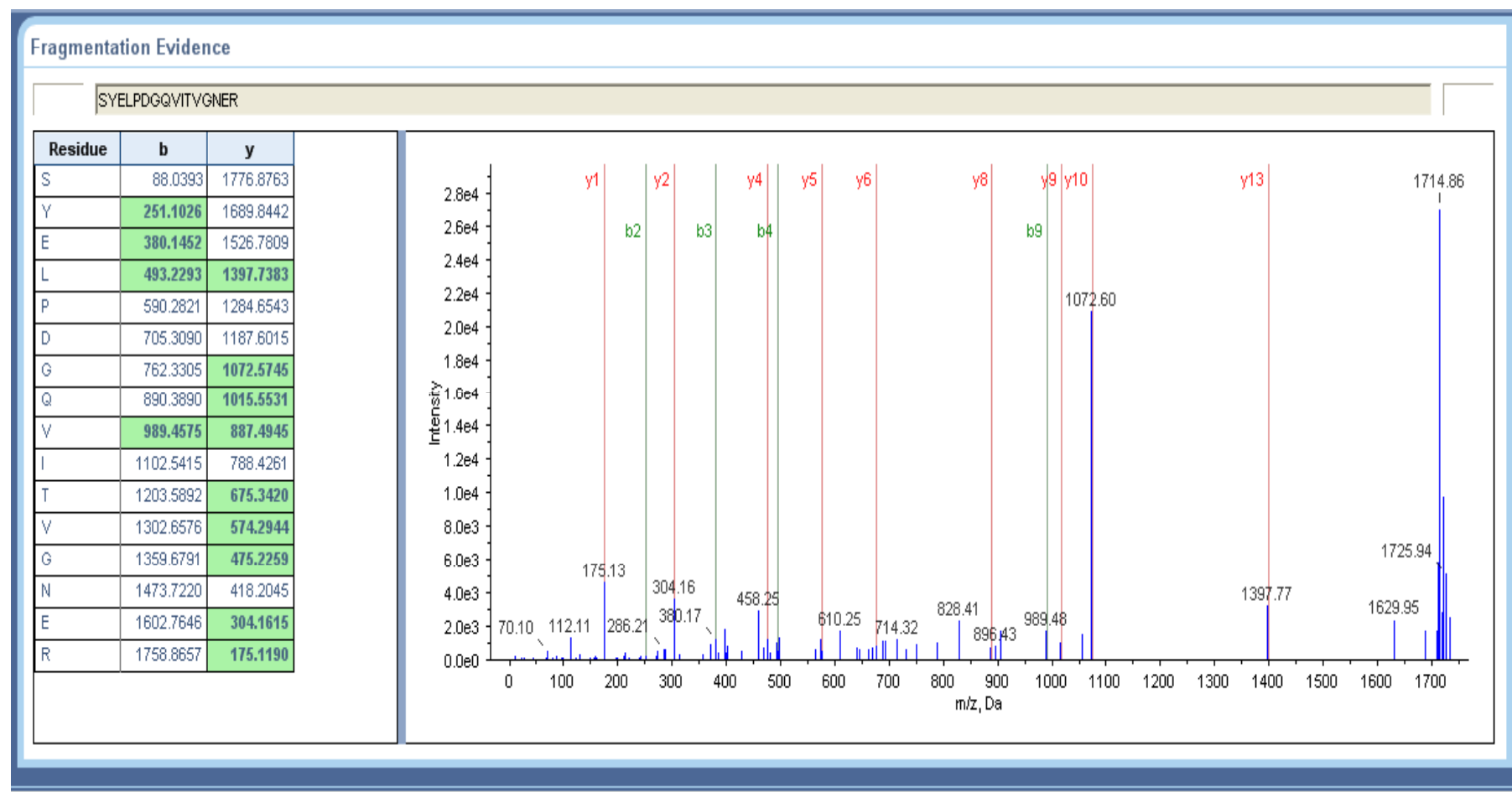

**Figure S1.** Screenshot of actin peptide obtained from ProteinPilot™

Supplement: Figure S1 — Screenshot of actin peptide obtained from ProteinPilotTM. (PDF) [file pone.0058781.s001.pdf]

**Figure S2.** The parent ion of actin. (A, B) The parent ion on the spectra (red arrows)

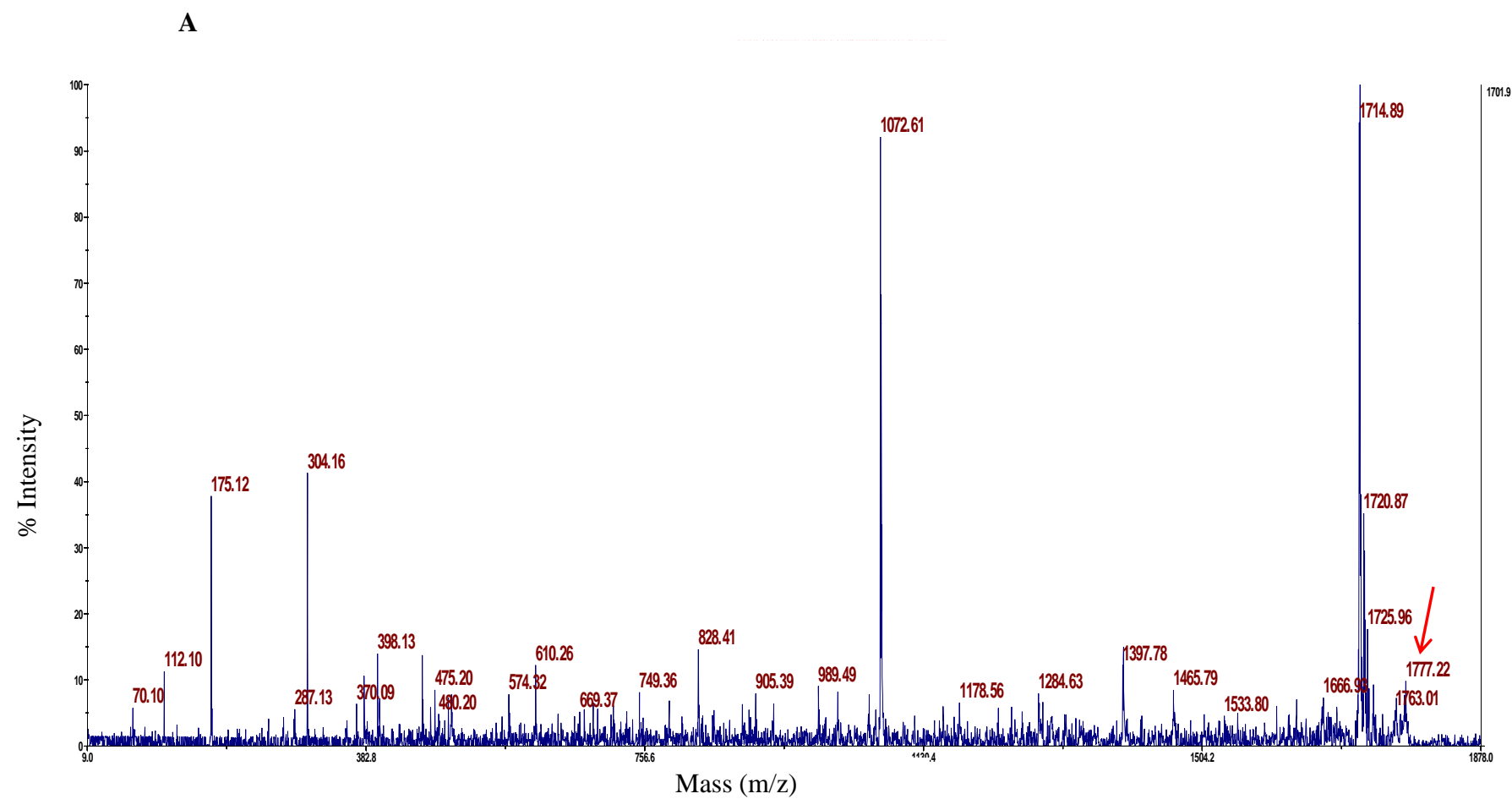

**B**

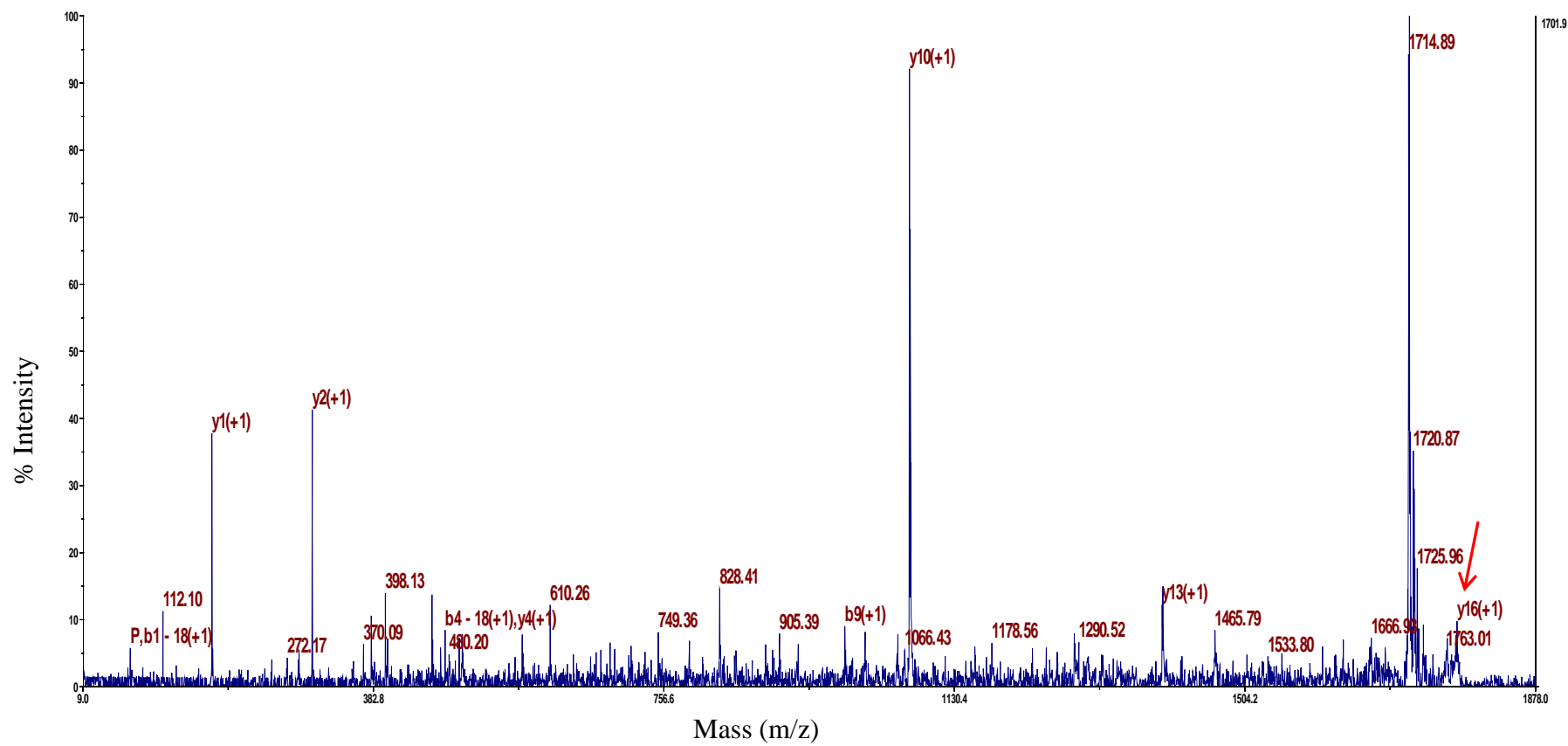

Supplement: Figure S2 — The parent ion of actin. (A, B) The parent ion on the spectra (red arrows). (PDF) [file pone.0058781.s002.pdf]

Fig. S3

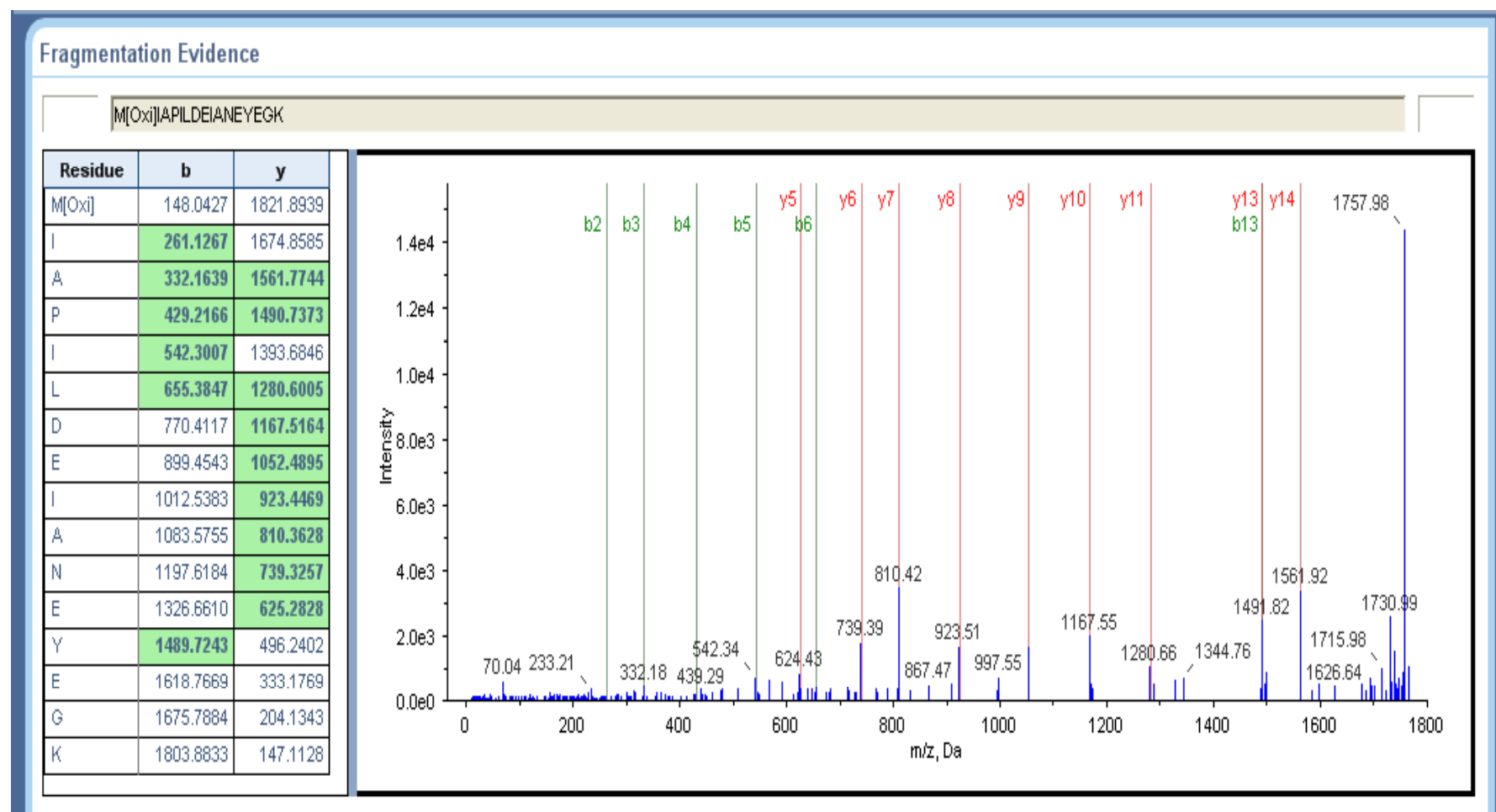

**Figure S3.** Screenshot of Thioredoxin peptide from ProteinPilot™

Supplement: Figure S3 — Screenshot of Thioredoxin peptide from ProteinPilotTM. (PDF) [file pone.0058781.s003.pdf]

Fig. S4

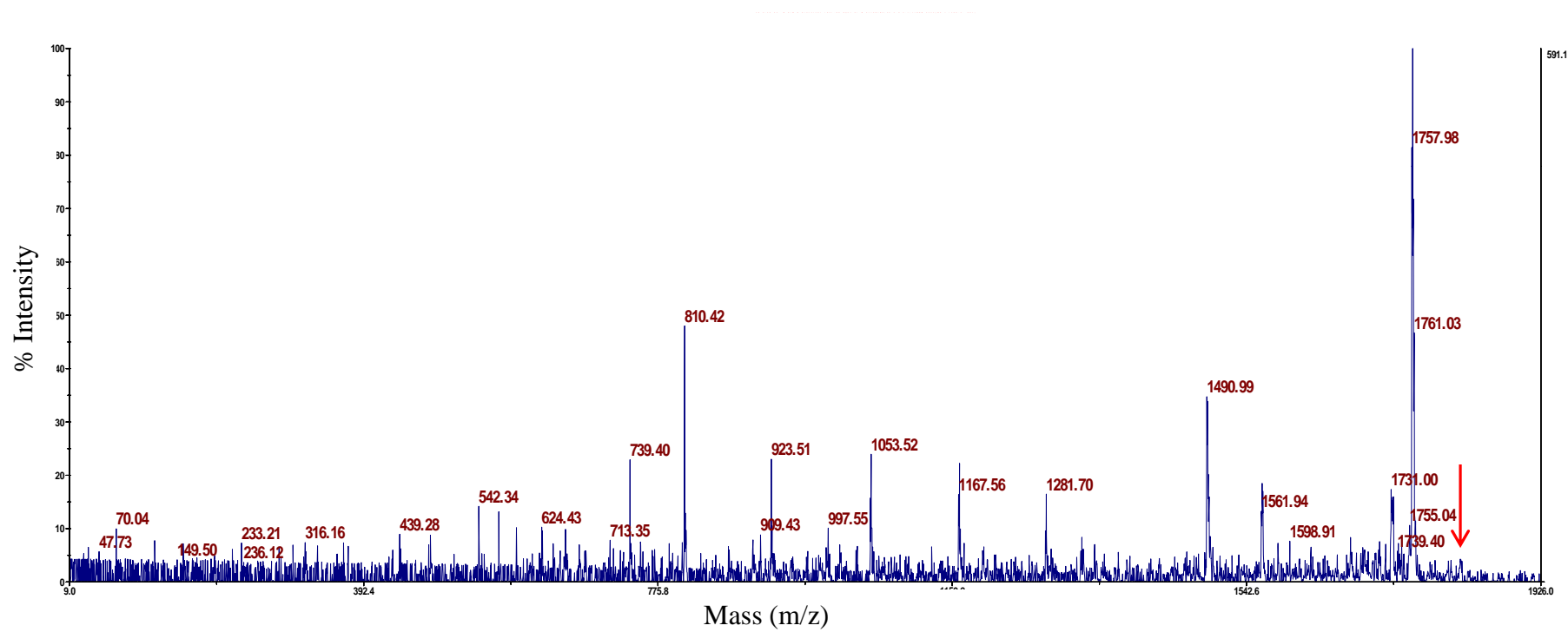

**Figure S4.** The parent ion on the spectra of Thioredoxin (red arrow)

Supplement: Figure S4 — The parent ion on the spectra of Thioredoxin (red arrow). (PDF) [file pone.0058781.s004.pdf]
