# Supplementary material for: Analysis of the Proteinaceous Components of the Organic Matrix of Calcitic Sclerites from the Soft Coral Sinularia sp
Source: PLoS One. 2013 Mar 14;8(3):e58781. doi: 10.1371/journal.pone.0058781 (PMC3597568; doi:10.1371/journal.pone.0058781)
Supplement: Table S1 — A list of proteins identified by de novo analysis from silver-stained SSCL-150 and highly conserved with other animals proteins. The proteins were obtained with 80–95% confidence, and the best hits were from the NCBI databases. All identifications were made by tandem MS/MS searches. (PDF) [file pone.0058781.s005.pdf]

Table S1. A list of proteins identified by *de novo* analysis from silver-stained SSCL-150 and highly conserved with other animals proteins. The proteins were obtained with 80-95% confidence, and the best hits were from the NCBI databases. All identifications were made by tandem MS/MS searches.

**No.**	**Identification**	**Number of Peptides**	**Accession. No. ⎸gi⎸**	**Species name** (best matched)
1	actin, putative	5	gi|67462785, gi|167394535, gi|183237603, gi|167380623, gi|88175361	*Entamoeba histolytica*, *E. dispar*, *Lygus lineolaris*
2	ACTin family member (act-4)	3	gi|71994099, gi|17568985, gi|17568987	*Caenorhabditis elegans*,
3	notochord actin	1	gi|8439515	*Branchiostoma belcheri*
4	Actin-1	8	gi|231494, gi|18314323, gi|170581695, gi|54778605, gi|40647174, gi|3907620, gi|2829755, gi|1168318	*Heterodera glycines*, *Rugia malayi*, *Sorosphaera veronicae*, *Penaeus monodon*,
5	Actin-2	5	gi|26421981, gi|170587336, gi|164454324, gi|113229, gi|81158087	*Globodera rostochiensis*, *Brugia malayi*, *Dicyema acuticephalum*, *Strongylocentrotus purpuratus*
6	Actin-3	1	gi|50593062	*Parastrongyloides trichosuri*
7	Actin, nonmuscle	1	gi|1703133	---
8	gamma-actin	1	gi|57013327	*Blakeslea trispora*
9	actin, gamma 1	1	gi|45361511	*Xenopus tropicalis*
10	Actin-6	1	gi|1703124	---
11	actin variant 1	1	gi|162280611	*Dictyocaulus viviparous*
12	beta-actin-1	1	gi|27475933	*Meloidogyne javanica*
13	beta-actin	19	gi|8886013, gi|33318285, gi|94537157, gi|7546805, gi|7546744, gi|63004272, gi|47550655, gi|33526989, gi|33318289, gi|33318287, gi|27805142, gi|161376754, gi|160693772, gi|160693770, gi|157382498, gi|157382496, gi|109716241, gi|10442729, gi|81174743,	*Oncorhynchus mykiss*, *Tigriopus japonicus* *Solea senegalensis* *Carassius auratus* *Rhynchocypris oxycephalus* *Platichthys flesus* *Cirrhinus molitorella*
14	beta-actin-like	1	gi|55741585	*Canislupus familiaris*
15	Actin, cytoplasmic 2 (Gamma-actin)	1	gi|82213656	---
16	Actin, cytoplasmic 1	1	gi|67462093	---
17	Actin, cytoplasmic 1 (Beta-actin-1)	1	gi|62298523	----

Table S1. (Continued)

18	beta cytoplasmic actin	1	gi|57977261	*Pagrus major*
19	actin, gamma 1 propeptide	1	gi|56119084	*Gallus gallus*
20	cytoskeletal actin IIIa	1	gi|47551039	*Strongylocentrotus purpuratus*
21	cytoskeletal actin CyIIIb	1	gi|47551035	*Strongylocentrotus purpuratus*
22	muscle-specific actin 3	1	gi|33642245	*Aedes aegypti*
23	Actin, muscle (BbMA1)	1	gi|3182904	---
24	Actin, muscle	1	gi|3182896	---
25	Actin, cytoskeletal 3A	1	gi|1703135	---
26	Actin, cytoskeletal 2A	1	gi|1703134	---
27	Actin, cytoplasmic 2 (Beta-actin B)	1	gi|1703111	---
28	fast muscle actin	1	gi|15321578	*Scyliorhinus retifer*
29	alpha actin	1	gi|15148888	*Homarus americanus*
30	cytoplasmic actin	5	gi|13699190, gi|89255272, gi|84682180, gi|84682178, gi|84682176	*Lethenteron japonicum Pinctada fucata, Hirudo medicinalis*
31	Actin, cytoplasmic 2 (Gamma-actin)	1	gi|82195535	---
32	b-actin	1	gi|7839450	*Oncorhynchus mykiss*
33	Alpha-Actin: Human Gelsolin Domain 1	1	gi|7766848	---
34	actin	111	gi|6628, gi|6626, gi|4235277, gi|33946359, gi|61655696, gi|53829584, gi|52352094, gi|163716525, gi|160337369, gi|12697142, gi|63099803, gi|63099799, gi|63099797, gi|63099793, gi|63099791, gi|41387730, gi|41387728, gi|41387726, gi|41387724, gi|2981073, gi|146218309, gi|146218305, gi|146218303, gi|139003933, gi|139003926, gi|12697526, gi|12697524, gi|12697522, gi|12697520, gi|12697518, gi|12697516, gi|12697514, gi|12697512, gi|12697510, gi|12697508, gi|12697506, gi|12697500, gi|12697308, gi|12697306, gi|12697304, gi|12697302, gi|12697298, gi|12697292, gi|12697290, gi|12697288, gi|12697284, gi|12697282, gi|12697280, gi|12697278, gi|12697276, gi|12697274, gi|12697266, gi|12697264, gi|12697262, gi|12697258,	*Amoeba proteus, Chaos carolinense, Entamoeba dispar Ciona intestinalis, Caenorhabditis elegans, Setaria digitata, Glaeseria mira Hartmannella cantabrigiensis, Panagrellus redivivus, Bursaphelenchus xylophilus, Heleopera sphagni, Corallochytrium limacisporum Heterodera cyperi, Ancyromonas sigmoides, Heterodera cyperi, Arcella hemisphaerica, Acropora millepora, Ancyromonas sp. Absidia glauca, Absidia spinosa, Absidia psychrophilia Absidia parricida, Absidia*

Table S1. (Continued)

			gi|12697256, gi|12697242, gi|12697234, gi|12697232, gi|12697228, gi|12697226, gi|12697216, gi|12697214, gi|12697212, gi|12697210, gi|12697181, gi|12697179, gi|12697177, gi|12697175, gi|12697173, gi|12697171, gi|12697169, gi|12697161, gi|12697159, gi|12697144, gi|125488969, gi|123316094, gi|123316090, gi|123316088, gi|123316086, gi|111610556, gi|111610550, gi|157125380, gi|9501243, gi|94468486, gi|77539277	*cylindrospora, Absidia californica, Heterodera ripae, Heterodera avenae, Heterodera litoralis, Heterodera schachtii, Heterodera latipons, Ostertagia ostertagi, Panagrolaimus superbus, Mucor mucedo Mucor plumbeus, Mucor flavus Rhizopus oryzae, Rhizopus oryzae, Zygorhynchus heterogamous,*
